# Brains of rhesus monkeys display Aβ deposits and glial pathology while lacking Aβ dimers and other Alzheimer's pathologies

**DOI:** 10.1111/acel.12978

**Published:** 2019-06-04

**Authors:** Jing Zhang, Baian Chen, Jing Lu, Yi Wu, Shubo Wang, Zitong Yao, Liming Zhu, Yanhua Qiao, Quan Sun, Wei Qin, Qiao Zhao, Jianping Jia, Cuibai Wei

**Affiliations:** ^1^ Innovation Center for Neurological Disorders Xuan Wu Hospital, Capital Medical University Beijing China; ^2^ Department of Neurobiology, School of Basic Medical Sciences Capital Medical University Beijing China; ^3^ Laboratory Animal Center Capital Medical University Beijing China; ^4^ Department of Neurobiology, Beijing Key Laboratory of Neural Regeneration and Repair Capital Medical University Beijing China; ^5^ Department of Neurology Xuan Wu Hospital, Capital Medical University Beijing China

**Keywords:** aging, Alzheimer's disease, amyloid beta (Aβ) deposits, Aβ oligomer, gliosis, tau pathology

## Abstract

Cerebral amyloid beta (Aβ) deposits are the main early pathology of Alzheimer's disease (AD). However, abundant Aβ deposits also occur spontaneously in the brains of many healthy people who are free of AD with advancing aging. A crucial unanswered question in AD prevention is why AD does not develop in some elderly people, despite the presence of Aβ deposits. The answer may lie in the composition of Aβ oligomer isoforms in the Aβ deposits of healthy brains, which are different from AD brains. However, which Aβ oligomer triggers the transformation from aging to AD pathogenesis is still under debate. Some researchers insist that the Aβ 12‐mer causes AD pathology, while others suggest that the Aβ dimer is the crucial molecule in AD pathology. Aged rhesus monkeys spontaneously develop Aβ deposits in the brain with striking similarities to those of aged humans. Thus, rhesus monkeys are an ideal natural model to study the composition of Aβ oligomer isoforms and their downstream effects on AD pathology. In this study, we found that Aβ deposits in aged monkey brains included 3‐mer, 5‐mer, 9‐mer, 10‐mer, and 12‐mer oligomers, but not 2‐mer oligomers. The Aβ deposits, which were devoid of Aβ dimers, induced glial pathology (microgliosis, abnormal microglia morphology, and astrocytosis), but not the subsequent downstream pathologies of AD, including Tau pathology, neurodegeneration, and synapse loss. Our results indicate that the Aβ dimer plays an important role in AD pathogenesis. Thus, targeting the Aβ dimer is a promising strategy for preventing AD.

## 1 INTRODUCTION

Alzheimer's disease (AD), the most common type of dementia, is a chronic and progressive neurodegenerative disease. Sporadic AD accounts for more than 95% of AD cases. Advancing age is the greatest risk factor for the development of sporadic AD (Guerreiro & Bras, 2015; Querfurth & LaFerla, 2010; Xia, Jiang, McDermott, & Han, 2018). The main pathologies of AD include abundant cerebral Aβ deposits, glia pathology, Tau pathology, neurodegeneration, and synapse loss. Among these features of AD, Aβ deposits occur very early and can lead to the downstream pathologies of AD (Selkoe & Hardy, 2016). However, abundant Aβ deposits also spontaneously develop in the brains of approximately 30% of healthy people with normal cognitive function during advancing aging (Rodrigue, Kennedy, & Park, 2009). The reason why not all people with Aβ deposits develop AD is an important unanswered question. Understanding the composition and pathological effects of Aβ deposits is the key to answering this question.

Aβ deposits are mainly comprised of monomers, oligomers, and fibrils. Convincing evidence strongly supports the amyloid cascade hypothesis (Selkoe & Hardy, 2016), which posits that Aβ oligomers in cerebral Aβ deposits are the main toxic molecules that trigger the subsequent downstream pathologies of AD, including glia pathology, Tau pathology, neurodegeneration, and synapse loss. In addition, Aβ oligomers are used as indicators to investigate the relationship between aging and the pathogenesis of AD, because Aβ oligomers participate in the aging process and the pathological transformation from aging to AD (Lesne et al., 2013). Due to the variety of Aβ oligomer lengths in Aβ deposits, including 2‐mer, 3‐mer, 5‐mer, 9‐mer, 10‐mer, 12‐mer oligomers, and others, the key Aβ oligomer that triggers the development of AD is controversial. Lesne et al. insist that 12‐mer Aβ oligomers, but not other oligomers, trigger AD pathologies (Amar et al., 2017; Lesne et al., 2006). In contrast, Shankar et al. argue that the Aβ dimer is the key molecule inducing Tau pathology, neurodegeneration, and synapse loss (Jin et al., 2011; Shankar et al., 2008). In these studies, Aβ oligomers were prepared in vitro from the brains of AD mice or patients and injected into rodents or used to treat cells, and the subsequent effects were analyzed. The limitations of these studies, which may have led to the discrepant results, include the following. First, Aβ oligomers readily self‐aggregate in vitro (Finder & Glockshuber, 2007; Hayden & Teplow, 2013), resulting in different responses in rodents and cells. Second, the high complexity of the Aβ oligomer extraction process leads to the presence of several artificial factors (Amar et al., 2017; Hayden & Teplow, 2013; Jin et al., 2011) that readily affect responses in rodents and cells. Third, Aβ oligomers extracted from the brains of AD mice or patients are exogenous stimuli for rodents and cells, and thus, they may have different effects compared to endogenous Aβ oligomers (Hayden & Teplow, 2013). Fourth, in contrast to the human brain, where Aβ deposits occur naturally with advancing age, Aβ deposits do not occur naturally in aging rodent brains (LaFerla & Green, 2012). All of these limitations could lead to erroneous conclusions about which Aβ oligomer triggers the downstream pathology of AD.

Nonhuman primates are phylogenetically close to humans, and Aβ sequences in several nonhuman primate species, including the rhesus monkey, cynomolgus monkey, and great ape, are identical to those found in humans (Heuer, Rosen, Cintron, & Walker, 2012). We and other researchers have shown that aged rhesus monkeys naturally develop Aβ deposits in the brain with striking similarities to those of aged humans (Heuer et al., 2012; Zhao et al., 2017). In addition, there is no need to prepare oligomers extracted from the Aβ deposits of AD mice or patients in vitro, because aged rhesus monkeys develop Aβ deposits naturally. Thus, the limitations of previous studies can be avoided by using rhesus monkey as a model for studying AD. Overall, aged rhesus monkeys are an ideal natural model for experiments studying the effects of endogenous Aβ oligomers from Aβ deposits in vivo.

In this study, we used rhesus monkeys that naturally develop Aβ deposits in the brain as a model. We analyzed the composition of Aβ oligomer isoforms in Aβ deposits and investigated the effects of natural Aβ deposits on AD pathologies in different regions of the brain, including the frontal cortex (FR), occipital cortex (OC), parietal cortex (PA), temporal cortex (TE), hippocampus (HI), midbrain (MI), thalamus (TH), and entorhinal cortex (EN). Our study provides clues that help to explain the transformation from aging to AD, and our findings may provide potential targets for preventing and treating AD.

## 2 RESULTS

### 2.1 Spontaneously developed cerebral Aβ deposits in aged rhesus monkey lack Aβ dimers

**Figure 1:**
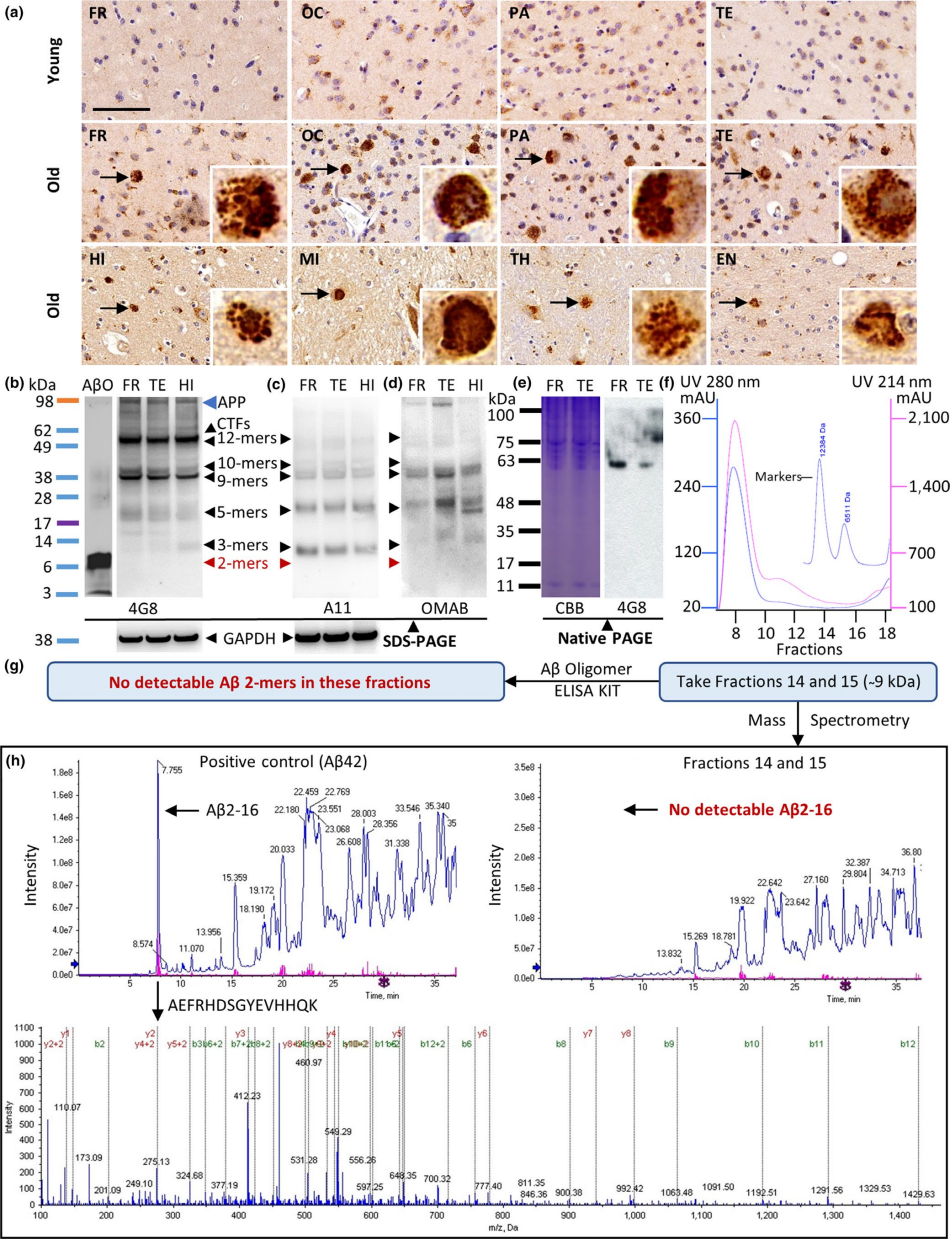
Cerebral Aβ deposits spontaneously developed in aged rhesus monkeys and contained oligomers, but lacked the Aβ dimers. (a) Representative photomicrographs of different brain regions from young and old rhesus monkeys, including the frontal cortex (FR), occipital cortex (OC), parietal cortex (PA), temporal cortex (TE), hippocampus (HI), midbrain (MI), thalamus (TH), and entorhinal cortex (EN). Brain sections were immunostained with a monoclonal anti‐Aβ antibody, 4G8, which recognizes Aβ deposits. The scale bar is 80 µm for all panels. The arrows indicate Aβ deposits. The areas indicated by the arrows are shown on the bottom right at four times higher magnification. (b–f) Brain homogenates were prepared from the FR, TE, and HI of old rhesus monkeys that were positive for Aβ deposits (a). The indicated homogenates were analyzed by SDS‐PAGE (b–d). The 4G8 monoclonal anti‐Aβ antibody that recognizes all Aβ forms, amyloid precursor protein (APP), and C‐terminal fragments (CTFs) was used in (b). The A11 polyclonal anti‐oligomer conformation‐specific antibody that can recognize Aβ oligomers was used in (c). The OMAB monoclonal antibody that specifically recognizes Aβ oligomers was used in (d). Protein molecular weight markers were used to estimate the molecular weights of Aβ oligomers. GAPDH was used as an internal loading control. (e) The indicated homogenates were analyzed by native PAGE, and stained by CBB and immunostained with 4G8 antibody. (f) The homogenates were fractionated using size‐exclusion chromatography (SEC). Aprotinin (6.511 kDa) and cytochrome C (12.384 kDa) were used as indicators of molecular size. Fractions 14 and 15 between 6.511 kDa and 12.384 kDa were collected (Aβ dimer are around 9 kDa). Aβ oligomers were analyzed in these fractions using an ELISA kit (g) and mass spectrometry (h). Synthetic Aβ42 was used as positive control in mass spectrometry analysis, and arrows indicate the peak of amino acid sequence from 2 to 16 of Aβ42 (AEFRHDSGYEVHHQK)

The amino acid sequence of Aβ in rhesus monkey is identical to that of the Aβ that spontaneously develops in human brains with advanced age (Heuer et al., 2012). We compared Aβ deposits in the brains of young and old rhesus monkeys using immunohistochemical staining with a monoclonal anti‐Aβ antibody that recognizes Aβ deposits (4G8). Aβ deposits were not observed in the cerebral cortex (including the FR, OC, PA, and TE) of young monkeys. However, five of eight old monkeys developed Aβ deposits in the cerebral cortex, including the FR, OC, PA, and TE (Figure 1a). In addition to the cerebral cortex, humans develop Aβ accumulations in the HI, MI, TH, and EN regions as they age (Hyman et al., 2012). Similar to humans, Aβ deposits were detected in the HI, MI, TH, and EN regions of old monkeys (Figure 1a).

To investigate the oligomer composition of Aβ deposits, Aβ oligomer isoforms from the FR, TE, and HI brain regions of old monkeys (Figure 1a) were analyzed by SDS‐PAGE and Western blotting. We used a monoclonal anti‐Aβ antibody (4G8) that recognizes all Aβ isoforms (Figure 1b). The immunoreactive protein bands detected at ~56, ~45, ~40, ~23, and ~14 kDa likely correspond to 12‐mer, 10‐mer, 9‐mer, 5‐mer, and 3‐mer Aβ oligomers, respectively (Figure 1b, arrowheads). There were no immunoreactive protein bands at ~9 kDa, which indicated a lack of SDS‐stable Aβ dimers (Figure 1b, red color arrowhead). The 4G8 antibody also recognizes the amyloid precursor protein (APP) and its C‐terminal fragments (CTFs). Thus, to confirm that the immunoreactive protein bands recognized by the 4G8 antibody were Aβ oligomers, rather than APP or CTFs, we used an anti‐oligomer conformation‐specific antibody A11 (Figure 1c) and an anti‐Aβ oligomer‐specific antibody OMAB (Figure 1d). The band around 98 kDa, which was detected by the 4G8 antibody in Figure 1b (indicated by blue arrowhead), was not detected by the A11 antibody, which recognizes the oligomer conformation rather than amino acid sequence (Figure 1c). Thus, the 98 kDa band is APP rather than an Aβ oligomer. The band between 49 and 62 kDa, which was detected by 4G8 in Figure 1b, contains Aβ 12‐mers and CTFs, because two Aβ oligomer antibodies A11 and OMAB are also able to detect this band but with less signal than the 4G8 antibody (Figure 1c and d). The immunoreactive protein bands detected at ~45, ~40, ~23, and ~14 kDa by 4G8, A11, and OMAB corresponded to 10‐mer, 9‐mer, 5‐mer, and 3‐mer Aβ oligomers, respectively (Figure 1b, c, and d, arrowheads). In agreement with the results in Figure 1b, there were no immunoreactive protein bands at ~9 kDa, which indicated a lack of SDS‐stable Aβ dimers (Figure 1c and d, red color arrowhead). The band at ~9 kDa is Aβ dimer and at ~4 kDa is Aβ monomer detected by 4G8 in Aβ oligomers (ABO) prepared by synthetic Aβ42 as a positive control (Figure 1b).

Researchers generally use SDS‐PAGE and Western blotting to analyze Aβ oligomers in human brain homogenates (Leinenga & Gotz, 2015; Lesne et al., 2013), but the native Aβ oligomers may not be identical to the SDS‐stable Aβ oligomers seen by SDS‐PAGE due to protein denaturation. Thus, we then analyzed the brain homogenates in native PAGE without protein denaturation. The Coomassie brilliant blue (CBB) staining and immunostaining with 4G8 did not detect a band at ~9 kDa (Figure 1e), in agreement with the results in Figure 1b–d. These data confirm a lack of Aβ dimers.

Evidence from cell and rodent model studies suggests that Aβ dimers are key toxic molecules that trigger subsequent downstream pathogenic AD events (Jin et al., 2011; Shankar et al., 2008). Thus, we confirmed the absence of Aβ dimers using two more sensitive methods. The Western blotting technique is able to detect Aβ at nanogram levels. However, sensitive enzyme‐linked immunosorbent assay (ELISA) kits are able to detect Aβ oligomers at picogram levels (as low as 4.41 pM). Unfortunately, ELISA kits are not able to distinguish between different lengths of Aβ oligomers (Fukumoto et al., 2010; Savage et al., 2014). Thus, we first fractionated the monkey brain homogenates based on molecular size using size‐exclusion chromatography (SEC). Using aprotinin (6.511 kDa) and cytochrome C (12.384 kDa) as markers, we collected fractions 14 and 15 for the detection of Aβ dimers (~9 kDa). No Aβ dimers in the indicated fractions of monkey brain were detected by ELISA (Figure 1g). To confirm the ELISA result, we further analyzed these fractions using mass spectrometry with high sensitivity and accuracy. Synthetic Aβ42 solution was used as a positive control. Mass spectrometry analysis shows a peak amino acid sequence from 2 to 16 of Aβ42 (AEFRHDSGYEVHHQK) in the positive control, but no peptides in the fractions of the monkey brain homogenates were identified by matching to the UniProt Macaca mulatta tcheliensis databases (Figure 1h). These data indicate a lack of Aβ dimers.

Taken all together, our results suggest that cerebral Aβ deposits spontaneously develop in aged rhesus monkeys, and these deposits contain Aβ oligomers but lack Aβ dimers.

### 2.2 Cerebral Aβ deposits lacking Aβ dimers induce glial pathology in vivo

**Figure 2:**
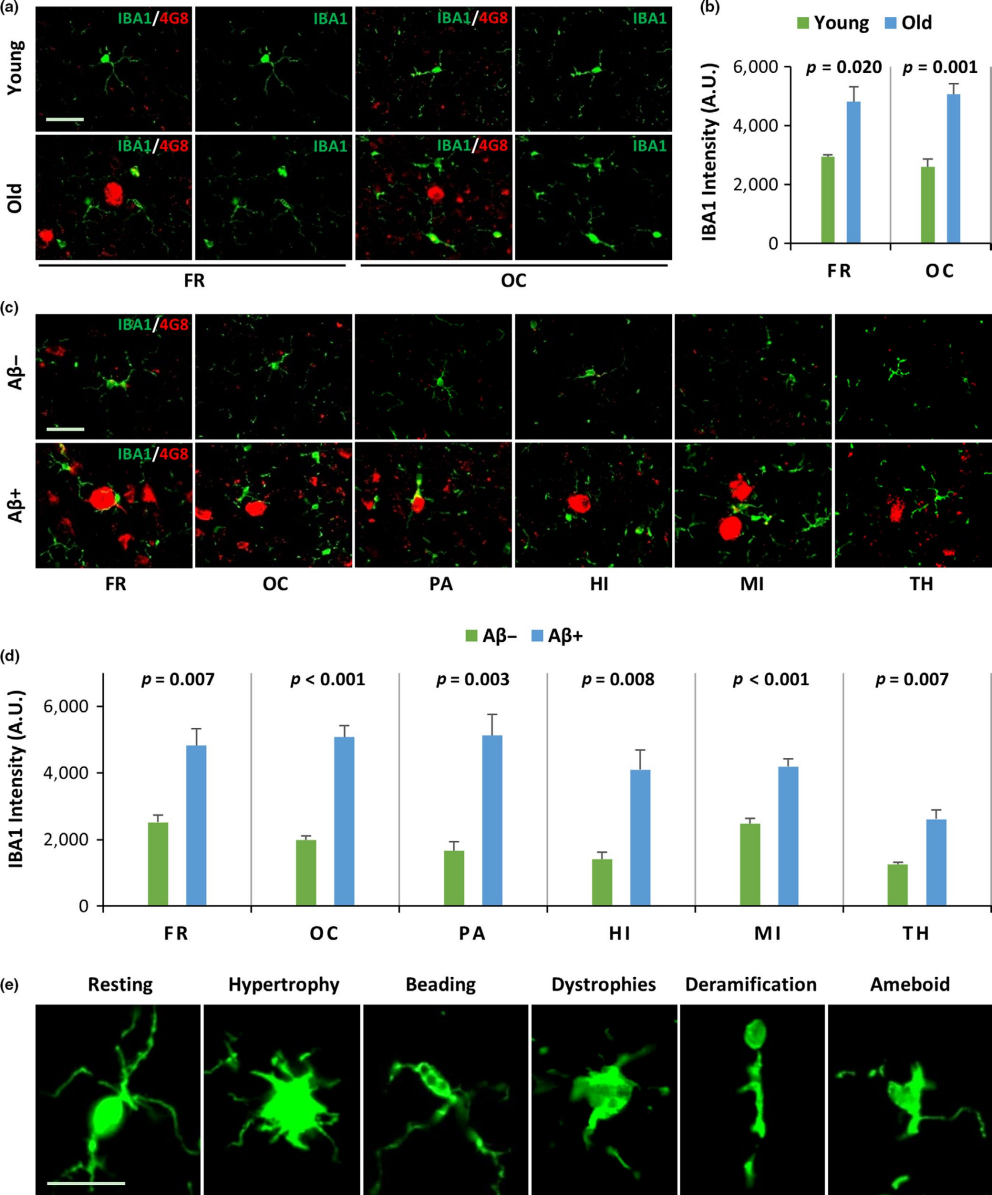
Cerebral Aβ deposits lacking Aβ dimers induce microglial pathologies in vivo. (a, b) The IBA1 intensity in the brains of old monkeys was increased in comparison with that of young monkeys. (a) Representative immunofluorescence confocal microscopy images of the frontal (FR) and occipital (OC) cortex regions from young and old rhesus monkeys. Continuous brain sections from Figure 1a were analyzed using double immunofluorescence staining (IBA1/4G8) with monoclonal anti‐Aβ antibodies (red) and anti‐IBA1 antibodies that label microglia (green) in the FR and OC of young and old monkeys. The scale bar is 40 µm for all panels. (b) Graphs show the quantification of the mean anti‐IBA1 (green) intensity from (a). The *p* values between young and old monkey brain were calculated using Student's *t* test. The error bars represent the *SEM* (*n* = 5/group). (c, d) The IBA1‐positive intensity was increased in areas with Aβ deposits (4G8) in old monkey brains. (c) Representative immunofluorescence confocal microscopy images of old monkey brains, including the frontal cortex (FR), occipital cortex (OC), parietal cortex (PA), hippocampus (HI), midbrain (MI), and thalamus (TH). Brain sections from old monkeys showing areas with no Aβ deposits (Aβ−) and areas with Aβ deposits (Aβ+) were double‐stained (IBA1/4G8) with monoclonal anti‐Aβ antibodies that recognize Aβ deposits (red) and anti‐IBA1 antibodies that recognize microglia (green). The scale bar is 40 µm for all panels. (d) Graphs show the quantification of the mean anti‐IBA1 (green) intensity from (c). The *p* values for the comparison of Aβ+ and Aβ− areas were calculated using Student's *t* test; the error bars represent the *SEM* (*n* = 5). (e) The morphological features of microglia were abnormal in areas of Aβ deposits in the brains of old monkeys. Representative high‐magnification images of IBA1‐positive microglia from Aβ− and Aβ+ areas in the brain of an old monkey. The microglia in Aβ− areas had normal morphology and were at rest, while the microglia in Aβ+ areas showed abnormal features, including hypertrophy, beading with spheroidal swellings, dystrophies, deramification, and ameboid morphology. The scale bar is 20 µm for all panels

**Figure 3:**
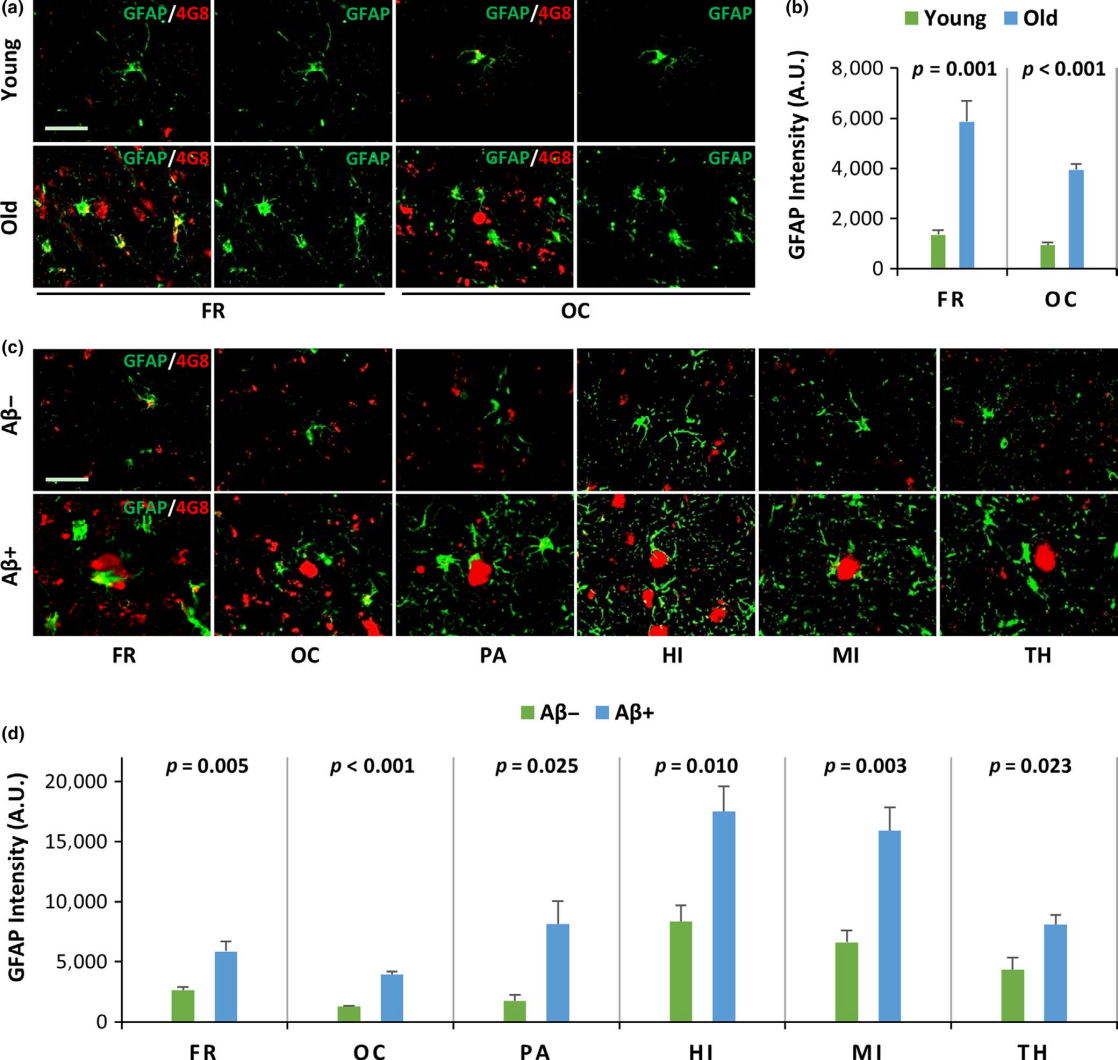
Cerebral Aβ deposits lacking Aβ dimers induce astrocytosis in vivo. (a, b) The GFAP intensity (astrocyte label) in young and old monkey brains is shown. (a) Representative immunofluorescence confocal microscopy images of the frontal (FR) and occipital (OC) cortex from young and old rhesus monkeys. Continuous brain sections from Figure 1a were analyzed using double immunofluorescence staining (GFAP/4G8) with monoclonal anti‐Aβ antibodies (4G8) that recognize Aβ deposits (red) and anti‐GFAP antibodies that recognize astrocytes (green) in the FR and OC regions of young and old monkeys. The scale bar is 40 µm for all panels. (b) The graphs show the quantification of the mean anti‐GFAP (green) intensity from (a). The *p* values between young and old monkeys were calculated using Student's *t* test; the error bars represent the *SEM* (*n* = 5). (c, d) The GFAP intensity was increased in areas with Aβ deposits in the brains of old monkeys. (c) Representative immunofluorescence confocal microscopy images of old monkey brains, including the frontal cortex (FR), occipital cortex (OC), parietal cortex (PA), hippocampus (HI), midbrain (MI), and thalamus (TH). Areas with no Aβ deposits (Aβ−) and areas with Aβ deposits (Aβ+) were double‐stained (GFAP/4G8) with monoclonal anti‐Aβ antibodies recognizing Aβ deposits (red) and anti‐GFAP antibodies recognizing astrocytes (green) in old monkeys. The scale bar is 40 µm for all panels. (d) The graphs show the quantification of the mean anti‐GFAP (green) intensity from (c); the *p* values between Aβ+ and Aβ− areas in the brains of old monkeys were calculated using Student's *t* test; the error bars represent the *SEM* (*n* = 5)

Previous studies reported that Aβ oligomers extracted from the cerebral deposits of mice and humans were able to induce glial pathology, including microglial pathology and astrocytosis, in cellular and murine models (Hu, Akama, Krafft, Chromy, & Eldik, 1998; Maezawa, Zimin, Wulff, & Jin, 2011). However, natural Aβ deposits do not occur in these models; thus, the effects of endogenous Aβ oligomers and deposits in vivo are unknown. To analyze the effects of spontaneously developing cerebral Aβ deposits on microglia and astrocytes, we analyzed the FR and OC regions of young and old monkeys using double immunofluorescence staining. Monoclonal anti‐Aβ antibodies (4G8) that recognize Aβ deposits were used in conjunction with anti‐IBA1 antibodies to label microglia or anti‐GFAP antibodies to label astrocytes. Immunopositive Aβ deposits were observed in the FR and OC of old monkeys, but not in those of young monkeys. The Aβ deposits were surrounded by many IBA1‐positive microglia (Figure 2a) or GFAP‐positive astrocytes (Figure 3a). The IBA1 intensity in the cerebral cortex of old monkeys was significantly higher than of young monkeys (*p* = 0.020 in FR, *p* = 0.001 in OC, Figure 2b). The GFAP intensity in the cerebral cortex of old monkeys was also significantly higher than that of young monkeys (*p* = 0.001 in FR, *p* < 0.001 in OC, Figure 3b). These results suggest that natural Aβ deposits in the brain during normal aging may induce gliosis (including microgliosis and astrocytosis) in monkeys.

Previous studies reported that age‐related factors may induce gliosis (Clarke et al., 2018; Jyothi et al., 2015; Spittau, 2017). Thus, we analyzed the effects of natural Aβ deposits on glia, including microglia and astrocytes, in old monkeys that were of similar ages to rule out the effects of age‐related factors on gliosis. We found that 4G8‐positive Aβ areas were closely surrounded by many IBA1‐positive microglia (Figure 2c) and GFAP‐positive astrocytes (Figure 3c) in the FR, OC, PA, HI, MI, and TH. The IBA1 intensity in areas with 4G8‐positive Aβ deposits in old monkeys was significantly higher than that observed in the areas with no Aβ deposits (*p* = 0.007 in FR, *p* < 0.001 in OC, *p* = 0.003 in PA, *p* = 0.008 in HI, *p* < 0.001 in MI, and *p* = 0.007 in TH, Figure 2d). We observed similar findings with GFAP; the GFAP intensity was significantly higher in areas with 4G8‐positive Aβ deposits in old monkeys in comparison with that of areas with no Aβ deposits (*p* = 0.005 in FR, *p* < 0.001 in OC, *p* = 0.025 in PA, *p* = 0.010 in HI, *p* = 0.003 in MI, and *p* = 0.023 in TH, Figure 3d). These results indicate that natural Aβ deposits, rather than age‐related factors, induce microgliosis and astrocytosis. In addition, microglia in areas with no Aβ deposits were predominant in the resting state. However, microglia in areas with 4G8‐positive Aβ deposits had morphological abnormalities, including hypertrophy, beading with spheroidal swelling, dystrophies, deramification, and ameboid morphology (Figure 2e). Taken together, our results suggest that cerebral Aβ deposits lacking Aβ dimers induce glial pathology in vivo, including microgliosis, abnormal microglia morphology, and astrocytosis.

### 2.3 Brains of rhesus monkeys lacking Aβ dimers did not show tau pathology

**Figure 4:**
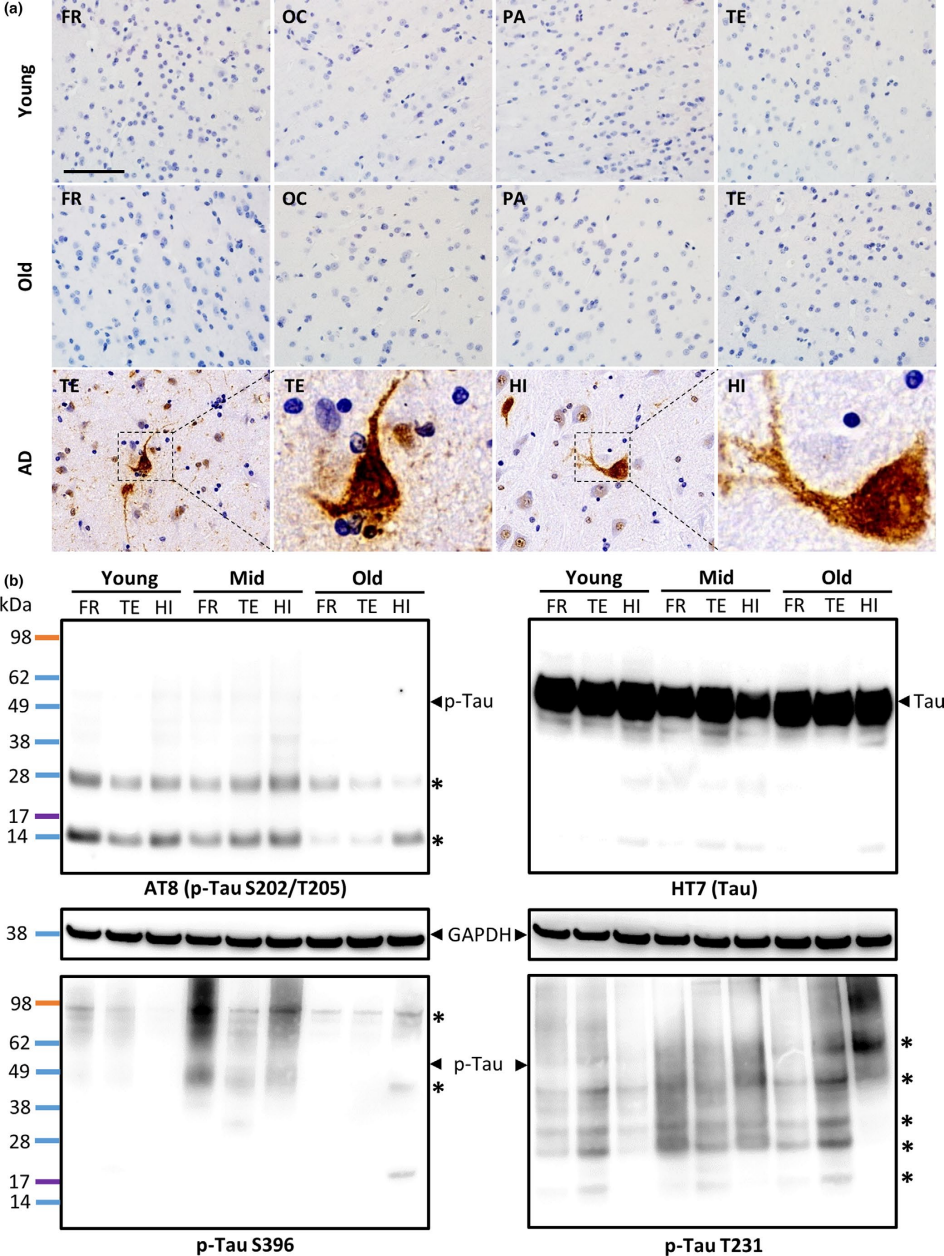
Brains of rhesus monkeys lacking Aβ dimers did not show tau pathology. (a) Representative photomicrographs of different brain regions (frontal cortex, FR; occipital cortex, OC; parietal cortex, PA; temporal cortex, TE) from young and old rhesus monkeys and an AD patient (temporal cortex, TE and hippocampus, HI). Continuous monkey brain sections from Figure 1a and sections from the brain of an AD patient were immunostained with monoclonal anti‐phospho‐Tau (p‐Tau) antibodies (AT8) that recognize phosphorylated Tau at serine residue 202 and threonine residue 205. The scale bar is 80 µm. The boxed areas (neurofibrillary tangles) in brain sections from the AD patient are shown at four times higher magnification. (b) Phospho‐Tau and total Tau were analyzed using Western blotting. Brain homogenates were prepared from the frontal cortex (FR), temporal cortex (TE), and hippocampus (HI) of rhesus monkeys at young, middle (Mid), and old age. Results obtained using a monoclonal antibody (AT8, p‐Tau S396, and p‐Tau T231) that recognizes p‐Tau are shown in the left and bottom panels, whereas results obtained using a monoclonal anti‐Tau antibody (HT7) that recognizes total Tau are shown in the right panel. Protein molecular weight markers (shown to the left of the gel) were used to estimate the molecular weights of p‐Tau and Tau. The asterisks (*) indicate nonspecific bands that were recognized by the AT8 antibody. GAPDH was used as an internal loading control

In cellular and murine models, Aβ oligomers instigate Tau pathology either directly or via glial pathology (Amar et al., 2017; De Felice et al., 2008; Jin et al., 2011). To determine whether spontaneously developed cerebral Aβ deposits induce Tau pathology during normal aging, we performed immunohistochemical staining with monoclonal anti‐p‐Tau antibodies (AT8) that recognize p‐Tau and neurofibrillary tangles (NFT). We stained continuous brain sections of young and old rhesus monkeys that showed Aβ deposits (Figure 1a). Immunopositive NFT staining was not detected in the cerebral cortex (including the FR, OC, PA, and TE) of young or old monkeys. neurofibrillary tangles‐positive staining was observed in positive control brain sections (TE and HI) from an AD patient (Figure 4a). In addition, no p‐Tau expression was detected by Western blotting with anti‐p‐Tau antibodies (AT8, p‐Tau S396, and p‐Tau T231) at the expected molecular weight of 55–62 kDa in brain homogenates (including the FR, TE, and HI) of young and old monkeys, although the normal form of Tau was detected at the expected size (Figure 4b). Taken together, our results indicate that the brains of rhesus monkeys lacking Aβ dimers did not show tau pathology.

### 2.4 Brains of rhesus monkeys lacking Aβ dimers did not show neurodegeneration, synapse loss, or working memory impairment

**Figure 5:**
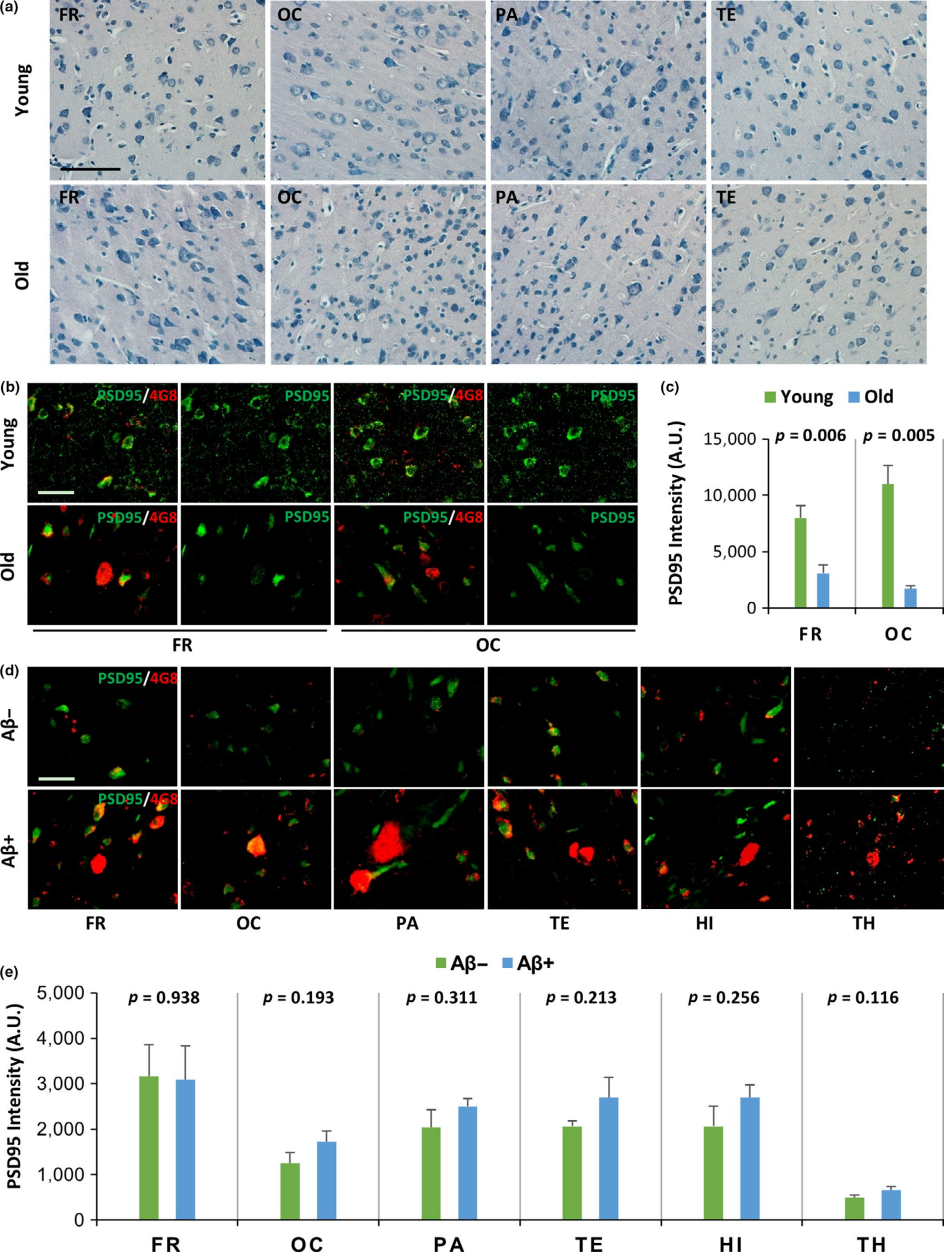
Brains of rhesus monkeys lacking Aβ dimers did not show neurodegeneration and synapse loss. (a) Representative photomicrographs of Nissl staining in different cerebral cortex areas (frontal cortex, FR; occipital cortex, OC; parietal cortex, PA; temporal cortex, TE) from young and old rhesus monkeys. The scale bar is 80 µm for all panels. (b) Representative immunofluorescence confocal microscopy images showing double immunofluorescence staining for Aβ deposits (4G8, red) and synapses (PSD95, green) in frontal (FR) and occipital (OC) cortex samples from young and old rhesus monkeys. The scale bar is 40 µm for all panels. (c) The graphs show the quantification of the mean anti‐PSD95 (green) intensity from (a). *p* Values between young and old monkey brains were calculated using Student's *t* test; the error bars represent the *SEM* (*n* = 5). (d) Representative immunofluorescence confocal microscopy images showing double immunofluorescence staining for Aβ deposits (4G8, red) and synapses (PSD95, green) in the frontal cortex (FR), occipital cortex (OC), parietal cortex (PA), temporal cortex (TE), hippocampus (HI), and thalamus (TH) of old monkeys. Areas with no Aβ deposits (Aβ−) and areas with Aβ deposits (Aβ+) were compared. The scale bar is 40 µm for all panels. (e) The graphs show the quantification of the mean anti‐PSD95 (green) intensity from (d). *p* Values comparing Aβ+ and Aβ− areas in the brains of old monkeys were calculated using Student's *t* test; the error bars represent the *SEM* (*n* = 5)

In cellular and murine models, Aβ oligomers generally induce neuron and synapse loss (Kim et al., 2003; Lacor et al., 2007; Shankar et al., 2008). To determine whether spontaneously developed cerebral Aβ deposits induce neuronal loss during normal aging, we performed Nissl staining on continuous brain sections of young and old rhesus monkeys containing Aβ deposits (Figure 1a). No degenerated neurons were observed in the cerebral cortex (including the FR, OC, PA, and TE) of young or old monkeys. This finding suggests that spontaneously developed cerebral Aβ deposits do not induce neurodegeneration in the brains of old rhesus monkeys (Figure 5a). To determine the effects of Aβ deposits on synapses during normal aging, we analyzed the cerebral cortex (FR and OC) of young and old monkeys using double immunofluorescence staining with monoclonal anti‐Aβ antibodies that recognize Aβ deposits and synapses (PSD95, a postsynapse marker). Synapse‐positive staining (PSD95) was observed in the cerebral cortices (FR and OC) of young and old monkeys (Figure 5b). The degree of synapse staining in the cerebral cortices of old monkeys was significantly lower than that of young monkeys (*p* = 0.006 in FR, *p* = 0.005 in OC, Figure 5b). Synapse staining (PSD95) in areas positive for Aβ deposits (4G8) did not differ significantly from that observed in areas negative for Aβ deposits (*p* = 0.938 in FR, *p* = 0.193 in OC, *p* = 0.311 in PA, *p* = 0.213 in TE, *p* = 0.256 in HI, and *p* = 0.116 in TH, Figure 5d,e). These results suggest that synapse loss occurs in the cerebral cortex with normal aging in monkeys. However, age‐related factors, rather than Aβ deposits, induce synapse loss in the brains of old monkeys. In addition, the working memory, tested using a delayed response task, was not significantly different between monkeys with Aβ deposits (Aβ+) and monkeys with no Aβ deposits (Aβ−) (*p* > 0.05), as shown in Figure S2. Taken together, our results indicate that brains of rhesus monkeys lacking Aβ dimers did not show neurodegeneration, synapse loss, or working memory impairment.

## 3 DISCUSSION

Currently, there are no effective treatments to stop or reverse AD. However, progress toward developing effective AD treatments may be greatly improved by clarifying the key molecules involved in the pathogenesis of AD. Previous reports suggest that Aβ oligomers in Aβ deposits are the main toxic isoforms that trigger the subsequent downstream pathogenic events associated with AD (Cline, Bicca, Viola, & Klein, 2018; Selkoe & Hardy, 2016). Since Aβ deposits are composed of Aβ oligomers with a variety of lengths, the Aβ oligomer that triggers the transformation from aging to AD is controversial. Previous researchers attempted to answer this question by analyzing the effects of various Aβ oligomers from mouse or human brains, which were injected into rodents or used to treat cells (Amar et al., 2017; Jin et al., 2011; Lesne et al., 2006; Shankar et al., 2008). Exogenous Aβ oligomers were prepared in vitro for these studies. In our study, we used rhesus monkeys, which develop cerebral Aβ deposits spontaneously with advancing age in a process thought to be similar to that which occurs in humans. Aβ deposits sometimes spontaneously occurred in the brains of cognitively healthy old humans and nondemented individuals with Alzheimer's disease neuropathology (Rodrigue et al., 2009; Zolochevska, Bjorklund, Woltjer, Wiktorowicz, & Taglialatela, 2018). We previously quantified the levels of insoluble Aβ42 in the brain of frontal cortex and temporal cortex of old monkeys (Zhao et al., 2017) and found that it was comparable to the levels of insoluble Aβ42 in healthy old human brains with Aβ deposits, but lower than the levels of insoluble Aβ42 in AD patient brains (Steinerman et al., 2008; Wang, Dickson, Trojanowski, & Lee, 1999). Thus, the rhesus monkey model used in our study is better than rodent models or cells, which do not develop Aβ deposits naturally.

We determined the oligomer composition of naturally developing Aβ deposits in the brains of rhesus monkeys, which was previously unknown (Heuer et al., 2012). SDS‐PAGE and Western blotting analyses using 4G8, a universal anti‐Aβ antibody, A11, a conformation‐dependent oligomer‐specific antibody, and OMAB, the Aβ oligomer‐specific monoclonal antibody, indicate that several Aβ oligomers develop in the monkey brain, including 3‐mer, 5‐mer, 9‐mer, 10‐mer, and 12‐mer oligomers. However, Aβ dimers were not observed. Our analysis of the oligomer composition of Aβ deposits using size‐exclusion chromatography accompanied by highly sensitive ELISAs and mass spectrometry confirmed the SDS‐PAGE and Western blotting data; Aβ dimers were not detected in old monkey brains. Researchers found several Aβ oligomers, including 3‐mer, 5‐mer, 9‐mer, 10‐mer, and 12‐mer oligomers using SDS‐PAGE and Western blotting, in aged human brains and AD patient brains, similar to our findings in monkey brain (Bao et al., 2012; Lesne et al., 2013). However, in contrast to monkey brains, cerebral Aβ dimers were detected in many patients with AD and some healthy elderly people, using the 4G8 antibody used in this study or different antibodies (Bao et al., 2012; Lesne et al., 2013; Shankar et al., 2008). Thus, although Aβ deposits in rhesus monkeys are similar to naturally occurring Aβ deposits in the human brain, they differ in one important aspect; monkey brain Aβ deposits lack Aβ dimers.

Researchers generally use SDS‐PAGE and Western blotting to analyze the Aβ oligomers, including the dimer, in human and mouse brain homogenates (Leinenga & Gotz, 2015; Lesne et al., 2013), but the SDS may affect the native Aβ oligomers like dimer in samples. Our results using native PAGE without SDS buffer treatment also indicate a lack of Aβ dimer. In addition, the samples without SDS pretreatment were analyzed using sensitive methods, including SEC combined‐ELISA and mass spectrometry, which also indicate a lack of Aβ dimer.

Aβ oligomers from Aβ deposits collected from AD patient brains or AD mouse brains induce glial pathology (Hu et al., 1998; Maezawa et al., 2011). However, the effects of spontaneously developed Aβ deposits devoid of Aβ dimers are unknown. We found that areas with Aβ deposits in the brains of old monkeys had significantly more gliosis, including microgliosis and astrocytosis, in comparison with young monkey brains or old monkey brains lacking Aβ deposits. Furthermore, microglia show resting normal morphology in areas lacking Aβ deposits, but they display a series of abnormal morphological features in areas with Aβ deposits. Abnormal morphological features of microglia include a hypertrophic shape, beading with spheroidal swellings, dystrophies, deramification, and ameboid morphology. Similar abnormal morphologies also occur in human AD brains with Aβ deposits (Sanchez‐Mejias et al., 2016). Our results provide strong evidence that spontaneously developed Aβ deposits in aged rhesus monkeys induce glial pathology in vivo, despite lacking Aβ dimers.

In addition to glial pathology, there are a series of downstream AD events, including Tau pathology, neurodegeneration, and synapse loss, that are necessary for the progression of AD (Selkoe & Hardy, 2016). Previous studies reported that Aβ oligomers extracted from Aβ deposits from mouse or human patient brains were able to trigger downstream AD pathogenic events (Cline et al., 2018; Selkoe & Hardy, 2016). In contrast to these studies, we found no differences in Tau pathology, neurodegeneration, or synapse loss in areas of brains with Aβ deposits in comparison with areas without Aβ deposits in monkey brains. These disparate results may be due to differences in the effects of exogenous Aβ oligomers in cellular and murine models in comparison with those of naturally occurring Aβ oligomers in monkeys. Aβ oligomers from humans are exogenous stimuli for cells and rodents. However, in our study, Aβ oligomers naturally developed in vivo with advanced age in monkeys.

Another reason for the differences in downstream effects in our study in comparison with those of other studies may be due to differences in Aβ oligomer sizes. Some controversy exists pertaining to which sizes of oligomers cause downstream AD pathology. Amar et al. showed that 12‐mer Aβ oligomers, but not 2‐mer or 3‐mer oligomers, triggered Tau pathology in rodents and a neuronal cell model (Amar et al., 2017). In contrast, other studies report that the Aβ dimer is the key molecule inducing Tau pathology, neurodegeneration, and synapse loss in rodents and neurons (Jin et al., 2011; Shankar et al., 2008). Our study shows that Aβ deposits with certain Aβ oligomers, including 3‐mer, 5‐mer, 9‐mer, 10‐mer, and 12‐mer oligomers, but not 2‐mer oligomers, did not trigger downstream AD pathogenic events. These results support the importance of the Aβ dimer in the progression of AD pathogenesis.

Tau is a microtubule‐associated protein that maintains neuronal cell shape, and age‐related Tau loss is thought to be related to neuronal loss (Avila, Barreda, Pallas‐Bazarra, & Hernandez, 2013). We detected Tau in the brains of monkeys at all ages, and its abundance did not change with age (Figure S1). These findings are consistent with a human study by Lesne et al. (Lesne et al., 2013). In our study, p‐Tau was undetectable in the brains of rhesus monkeys at all ages. In contrast, p‐Tau has been detected in the brains of aged humans and nondemented individuals with Alzheimer's disease neuropathology (Braak, Thal, Ghebremedhin, & Del, 2011; Zolochevska et al. 2016). Oikawa et al. also found that p‐Tau is occasionally detected in the brains of very old cynomolgus monkeys (older than 32 years of age) (Oikawa, Kimura, & Yanagisawa, 2010). Our results demonstrated no significant differences in PSD95 between the Aβ+ and Aβ− groups of old monkey brains. These data are consistent with human studies showing that the brains of cognitively healthy old humans and nondemented individuals with Alzheimer's disease neuropathology had Aβ deposits but did not show synapse loss (Selkoe & Hardy, 2016; Zolochevska et al., 2018). The lack of change in the abundance of Tau and p‐Tau in monkeys may partially explain the lack of synapse and neuronal loss and AD development in aged monkeys (Heuer et al., 2012). There are four amino acids in the rhesus monkey Tau that are different from human Tau. These amino acids are the product of the tau gene exon 8, which may prevent the formation of p‐Tau leading to a lack of Tau pathology in the rhesus monkey (Heuer et al., 2012).

In summary, we showed that rhesus monkeys, which are phylogenetically close to humans, naturally developed cerebral Aβ deposits. The Aβ deposits included 3‐mer, 5‐mer, 9‐mer, 10‐mer, and 12‐mer oligomers, but not Aβ dimer. The natural Aβ deposits, which were devoid of Aβ dimers, were able to induce gliosis, but did not trigger subsequent downstream AD pathogenic events. Our results support the hypothesis that the Aβ dimer is a key upstream molecule promoting the pathogenic progression of AD. Thus, blocking the accumulation of Aβ dimers may be an effective strategy for inhibiting the progression of AD.

## 4 EXPERIMENTAL PROCEDURES

### 4.1 Animals and sample preparation

Rhesus monkeys (*Macaca mulatta*) were purchased from the Beijing Institute of Xieerxin Biology Resource (Beijing, China) and the Academy of Military Medical Laboratory Animal Center (Beijing, China). The animals were housed at the Laboratory Animal Center of Capital Medical University (Beijing, China) according to the “Guidelines for the Care and Use of Laboratory Animals” and established in‐house protocols. Each animal had a routine health checkup to ensure that none suffered from neurological or other disorders or injuries. The monkeys in the young group were in the age range of 5–9 years (*n* = 5, four males and one female). The monkeys in the middle group were in the age range of 10–15 years (*n* = 3, one male and two females). The monkeys in the old group ranged in age from 19 to 31 years. Five of eight animals in the old monkey group spontaneously developed Aβ cerebral deposits (*n* = 5, two males and three females). Samples from different brain regions, including the FR, OC, PA, TE, HI, MI, TH, and EN, were harvested from monkeys after an overdose of anesthetics (8 mg/kg ketamine, followed by 50 mg/kg pentobarbital sodium). A portion of each tissue sample was fixed with 4% paraformaldehyde solution to allow the preparation of paraffin sections for immunohistochemical and immunofluorescent analyses. For biochemical assays, including Western blotting, SEC, and enzyme‐linked immunosorbent assays (ELISAs), a portion of each tissue sample was immediately frozen in liquid nitrogen and stored at −80°C until use. The brain sections of AD patients were generously gifted by Prof. Mengchao Cui of Beijing Normal University (China).

### 4.2 Immunohistochemistry and immunofluorescence

Immunohistochemical analyses were performed as described previously (Forny‐Germano et al., 2014; Zhao et al., 2017). Briefly, the fixed brain tissues were continuously cross‐sectioned in 4 μm sections and preserved in 0.1 M phosphate buffer (pH 7.6) at 4°C until use. For single‐label light microscopic analysis, the sections were incubated with the primary antibody, including the anti‐Aβ antibody (4G8, 1:500, Cat. No. 800701, BioLegend, San Diego, CA, USA) and the monoclonal anti‐p‐Tau antibody (AT8, 1:200, Cat. No. MN1020, Thermo Fisher Scientific), overnight at 4°C. After washing, the cross sections were incubated with a species‐compatible horseradish peroxidase‐conjugated secondary antibody (1:100; Cat. No. SPN‐9002, ZSGB‐BIO, Beijing, China) for 1 hr, followed by color development with diaminobenzidine. For double‐labeling immunofluorescence analysis, the sections were incubated with the anti‐Aβ antibody (4G8, 1:600) and the anti‐IBA1 antibody (1:300, Abcam, catalog #ab178847), the anti‐GFAP antibody (1:800, Abcam, catalog #ab7260), or the anti‐PSD95 antibody (1:300, Abcam, catalog #ab18258). The secondary antibodies were Alexa Fluor 488 (1:3,000, Abcam, catalog #150077) and Alexa Fluor 647 (1:3,000, Abcam, catalog #150155). The sections were observed under a 3DHISTECH slide scanner (Pannoramic MIDI, Budapest, Hungary).

### 4.3 Nissl staining

For Nissl staining, the brain sections were deparaffinized, soaked in 0.5% toluidine blue solution at room temperature for 10 min, and then rinsed with distilled water. Next, the brain sections were differentiated in 0.5% glacial acetic acid, dehydrated, cleared, and mounted.

### 4.4 Image analysis

The intensity of immunoreactive IBA‐1, GFAP, and PSD‐95 in each section of the monkey brain was quantified from five representative images using Fiji (www.fiji.sc).

### 4.5 SDS‐PAGE, native PAGE, and Western blotting analysis

The abundance of Aβ oligomers, Tau, and p‐Tau in brain homogenates samples was measured by SDS‐PAGE and Western blotting analysis. The abundance of Aβ oligomers was measured by native PAGE and Western blotting analysis. Homogenates from the FR, TE, and HI brain areas of young and old rhesus monkeys were prepared in PBS containing broad‐spectrum protease inhibitors at a tissue:buffer ratio of 1:20. The SDS‐PAGE was performed as follows: The homogenates were combined with sample buffer with SDS (Cat. No. NP0007, Invitrogen, Beijing, China) and loaded onto 4%–12% Bis‐Tris gels (Cat. No. NP0322BOX, Invitrogen) for electrophoresis. The native PAGE was performed as follows: 100 µg of total protein was prepared and subjected to native PAGE 4%–16% Bis‐Tris gel electrophoresis. Tris‐glycine buffer without SDS was used as a running buffer. Native‐Mark unstained protein standards were used as molecular weight markers. After native gel electrophoresis, acrylamide gels were rinsed in purified water and then stained with Coomassie brilliant blue. In addition, the separated proteins by SDS‐PAGE and native PAGE were then transferred to polyvinylidene fluoride membranes. To quantify target proteins, the membranes were blocked with skim milk and then incubated with the indicated primary antibodies overnight at 4°C. The monoclonal anti‐Aβ antibody (4G8, 1:1,000) reacted with amino acid residues 17–24 of Aβ. The oligomer A11 polyclonal antibody (1:1,000, Cat. No. AHB0052, Thermo Fisher Scientific, Waltham, MA, USA) is a conformation‐dependent antibody, which recognizes oligomers assembled from Aβ and α‐synuclein, rather than their amino acid sequences. The OMAB antibody is an anti‐Aβ oligomer‐specific antibody (1:1,000, AS10 932, Agrisera, Sweden). The monoclonal anti‐Tau antibody (HT7, 1:1,000, Cat. No. MN1000, Thermo Fisher Scientific) reacted with amino acid residues 159–163 of tau. The monoclonal anti‐p‐Tau antibody (AT8, 1:500, Cat. No. MN1020, Thermo Fisher Scientific) reacted with the phosphorylated amino acid residues Ser202/Thr205 of tau. Additional Tau antibodies included monoclonal anti‐p‐Tau S396 and p‐Tau T231 antibodies (1:10,000, p‐Tau S396, Cat. No. ab109390; 1:1,000, p‐Tau T231, Cat. No. ab151559; Abcam, Beijing, China). A polyclonal anti‐GAPDH antibody (Cat. No. ab9485, Abcam, Beijing, China) served as the internal control. Thereafter, the membranes were washed and incubated with secondary antibodies for 1 hr. The immunoreactive protein bands were visualized using the ECL Plus Western Blotting Detection System (Cat. No. RPN2132, GE Healthcare, Beijing, China) and quantified by densitometry using a UVItec Cambridge Imaging System (UVItec Limited, Cambridge, United Kingdom). The immunoreactive protein bands were scanned and expressed as densitometric light units.

### 4.6 SEC assay

Brain homogenates were centrifuged and filtered through 0.22‐µm filters to remove nuclei and large debris. Then, the supernatant (0.5 ml) was chromatographed on a Superdex 75 10/300 GL column using an AKTA purifier (GE Healthcare, Uppsala, Sweden) at a flow rate of 0.5 ml/min with 50 mM ammonium acetate (pH 8.5). The corresponding fractions were collected according to the previously reported method without the immunodepletion step (Shankar et al., 2008).

### 4.7 Measurement of Aβ dimer in the SEC fractions by Aβ oligomer ELISA

Aβ oligomers in the 6–12 kDa SEC fractions were detected using a commercially available ELISA kit (Cat. No. 27725, IBL Co., Ltd., Gunma, Japan) according to the manufacturer's instructions. The sensitivity of this kit was as low as 4.41 pM.

### 4.8 Mass spectrometry analysis

Predigested samples (2 µl) were injected onto a C18 desalted column (3 µm, 120Å, 350 µm × 0.5 mm) and separated using a C18 analysis column (3 µm, 120Å, 75 µm × 150 mm) with a gradient from 5% to 16% buffer B for 25 min, from 16% to 26% buffer B for 20 min, from 26% to 40% buffer B for 3 min, from 40% to 80% buffer B for 5 min, and 80% to 5% buffer B for 7 min, for total of 60 min at a flow rate of 0.6 μl/min. Peptides present were identified by matching to the UniProt Macaca mulatta tcheliensis databases. The Paragon algorithm in Protein Pilot v 5.0 (SCIEX) was used to search the databases. Databases using the following search parameters were used: trypsin used for digestion, the state of cysteine alkylation, the “ID focus” which was typically “Biological modifications,” and the type of “Search effort” which was typically “Thorough.” Data acquisition was performed with a Triple‐TOF 6600 mass spectrometer (Sciex, USA) fitted with a Nanospray III source (Sciex, USA). The ion spray voltage was 2,300 V, declustering potential 80 V, curtain gas 35 psi, nebulizer gas 5 psi, and interface heater temperature 150°C. Peptides were introduced into the mass spectrometer using Nona 415 liquid chromatography (Sciex, USA). Chromatography solvents were water/acetonitrile/formic acid (98/2/0.1%, B 2/98/0.1%).

### 4.9 Test monkeys' working memory using a delayed response task

The procedures for testing monkeys' working memory were performed in a Wisconsin General Testing Apparatus using the method previously reported (Li et al., 2010).

### 4.10 Statistical analysis

All data were analyzed using spss 20.0 software (IBM, Inc., Armonk, NY, USA). The intensity of immunoreactive IBA‐1, GFAP, and PSD‐95 (between different brain regions of young and old monkeys or between Aβ+ and Aβ− areas in old monkeys) was compared using Student's *t* test. The densitometric light units of the Tau in each selected region of the monkey brain were compared at three different ages using one‐way ANOVA, followed by Tukey's post hoc test. A *p*‐value < 0.05 was considered to indicate statistical significance.

## CONFLICT OF INTEREST

None declared.

## AUTHORS' CONTRIBUTION

CW, JZ, BC, JP and JL designed the study and analyzed the data. CW, JZ, and BC wrote the manuscript. JZ, BC, SW, ZY, YQ, and QZ performed the experiments. YW, QS, and WQ provided biochemical and morphological technical analysis and assistance.

## References

[acel12978-bib-0001] Amar, F. , Sherman, M. A. , Rush, T. , Larson, M. , Boyle, G. , Chang, L. , … Lesne, S. E. (2017). The amyloid‐β oligomer Aβ*56 induces specific alterations in neuronal signaling that lead to tau phosphorylation and aggregation. Science Signalling, 10, eaal2021 10.1126/scisignal.aal2021 PMC5859319 28487416

[acel12978-bib-0002] Avila, J. , de Barreda, E. G. , Pallas‐Bazarra, N. , & Hernandez, F. (2013). Tau and Neuron Aging. Aging and Disease, 4, 23–28. 23423462 PMC3570138

[acel12978-bib-0003] Bao, F. , Wicklund, L. , Lacor, P. N. , Klein, W. L. , Nordberg, A. , & Marutle, A. (2012). Different beta‐amyloid oligomer assemblies in Alzheimer brains correlate with age of disease onset and impaired cholinergic activity. Neurobiology of Aging, 33, 821–825. 10.1016/j.neurobiolaging.2011.05.003 21683475

[acel12978-bib-0004] Braak, H. , Thal, D. R. , Ghebremedhin, E. , & Del, T. K. (2011). Stages of the pathologic process in Alzheimer disease: Age categories from 1 to 100 years. Journal of Neuropathology and Experimental Neurology, 70, 960–969. 10.1097/NEN.0b013e318232a379 22002422

[acel12978-bib-0005] Clarke, L. E. , Liddelow, S. A. , Chakraborty, C. , Munch, A. E. , Heiman, M. , & Barres, B. A. (2018). Normal aging induces A1‐like astrocyte reactivity. Proceedings of the National Academy of Sciences of the United States of America, 115, E1896–E1905. 10.1073/pnas.1800165115 29437957 PMC5828643

[acel12978-bib-0006] Cline, E. N. , Bicca, M. A. , Viola, K. L. , & Klein, W. L. (2018). The amyloid‐beta oligomer hypothesis: Beginning of the third decade. Journal of Alzheimer's Disease, 64, S567–S610. 10.3233/JAD-179941 PMC6004937 29843241

[acel12978-bib-0007] De Felice, F. G. , Wu, D. , Lambert, M. P. , Fernandez, S. J. , Velasco, P. T. , Lacor, P. N. , … Klein, W. L. (2008). Alzheimer's disease‐type neuronal tau hyperphosphorylation induced by a beta oligomers. Neurobiology of Aging, 29, 1334–1347. 10.1016/j.neurobiolaging.2007.02.029 17403556 PMC3142933

[acel12978-bib-0008] Finder, V. H. , & Glockshuber, R. (2007). Amyloid‐beta Aggregation. Neuro‐degenerative Diseases, 4, 13–27. 10.1159/000100355 17429215

[acel12978-bib-0009] Forny‐Germano, L. , Lyra, E. S. N. , Batista, A. F. , Brito‐Moreira, J. , Gralle, M. , Boehnke, S. E. , … De Felice, F. G. (2014). Alzheimer's disease‐like pathology induced by amyloid‐beta oligomers in nonhuman primates. The Journal of Neuroscience, 34, 13629–13643. 10.1523/JNEUROSCI.1353-14.2014 25297091 PMC6608380

[acel12978-bib-0010] Fukumoto, H. , Tokuda, T. , Kasai, T. , Ishigami, N. , Hidaka, H. , Kondo, M. , … Nakagawa, M. (2010). High‐molecular‐weight beta‐amyloid oligomers are elevated in cerebrospinal fluid of Alzheimer patients. The FASEB Journal, 24, 2716–2726. 10.1096/fj.09-150359 20339023

[acel12978-bib-0011] Guerreiro, R. , & Bras, J. (2015). The age factor in Alzheimer's disease. Genome Medicine, 7, 106 10.1186/s13073-015-0232-5 26482651 PMC4617238

[acel12978-bib-0012] Hayden, E. Y. , & Teplow, D. B. (2013). Amyloid beta‐protein oligomers and Alzheimer's disease. Alzheimer's Research and Therapy, 5, 60 10.1186/alzrt226 PMC3978746 24289820

[acel12978-bib-0013] Heuer, E. , Rosen, R. F. , Cintron, A. , & Walker, L. C. (2012). Nonhuman primate models of Alzheimer‐like cerebral proteopathy. Current Pharmaceutical Design, 18, 1159–1169. 22288403 10.2174/138161212799315885 PMC3381739

[acel12978-bib-0014] Hu, J. , Akama, K. T. , Krafft, G. A. , Chromy, B. A. , & Van Eldik, L. J. (1998). Amyloid‐beta peptide activates cultured astrocytes: Morphological alterations, cytokine induction and nitric oxide release. Brain Research, 785, 195–206. 9518610 10.1016/s0006-8993(97)01318-8

[acel12978-bib-0015] Hyman, B. T. , Phelps, C. H. , Beach, T. G. , Bigio, E. H. , Cairns, N. J. , Carrillo, M. C. , … Montine, T. J. (2012). National Institute on Aging‐Alzheimer's association guidelines for the neuropathologic assessment of Alzheimer's disease. Alzheimer's and Dementia: The Journal of the Alzheimer's Association, 8, 1–13. 10.1016/j.jalz.2011.10.007 PMC3266529 22265587

[acel12978-bib-0016] Jin, M. , Shepardson, N. , Yang, T. , Chen, G. , Walsh, D. , & Selkoe, D. J. (2011). Soluble amyloid beta‐protein dimers isolated from Alzheimer cortex directly induce Tau hyperphosphorylation and neuritic degeneration. Proceedings of the National Academy of Sciences of the United States of America, 108, 5819–5824. 10.1073/pnas.1017033108 21421841 PMC3078381

[acel12978-bib-0017] Jyothi, H. J. , Vidyadhara, D. J. , Mahadevan, A. , Philip, M. , Parmar, S. K. , Manohari, S. G. , … Alladi, P. A. (2015). Aging causes morphological alterations in astrocytes and microglia in human substantia nigra pars compacta. Neurobiology of Aging, 36, 3321–3333. 10.1016/j.neurobiolaging.2015.08.024 26433682

[acel12978-bib-0018] Kim, H.‐J. , Chae, S.‐C. , Lee, D.‐K. , Chromy, B. , Lee, S. C. , Park, Y.‐C. , … Hong, S.‐T. (2003). Selective neuronal degeneration induced by soluble oligomeric amyloid beta protein. The FASEB Journal, 17, 118–120. 10.1096/fj.01-0987fje 12424218

[acel12978-bib-0019] Lacor, P. N. , Buniel, M. C. , Furlow, P. W. , Clemente, A. S. , Velasco, P. T. , Wood, M. , … Klein, W. L. (2007). Aβ oligomer‐induced aberrations in synapse composition, shape, and density provide a molecular basis for loss of connectivity in Alzheimer's disease. Journal of Neuroscience, 27, 796–807. 10.1523/JNEUROSCI.3501-06.2007 17251419 PMC6672917

[acel12978-bib-0020] LaFerla, F. M. , & Green, K. N. (2012). Animal models of Alzheimer disease. Cold Spring Harbor Perspectives in Medicine, 2, 10.1101/cshperspect.a006320 PMC3543097 23002015

[acel12978-bib-0021] Leinenga, G. , & Gotz, J. (2015). Scanning ultrasound removes amyloid‐beta and restores memory in an Alzheimer's disease mouse model. Science Translational Medicine, 7, 278ra33 10.1126/scitranslmed.aaa2512 25761889

[acel12978-bib-0022] Lesne, S. , Koh, M. T. , Kotilinek, L. , Kayed, R. , Glabe, C. G. , Yang, A. , … Ashe, K. H. (2006). A specific amyloid‐beta protein assembly in the brain impairs memory. Nature, 440, 352–357. 10.1038/nature04533 16541076

[acel12978-bib-0023] Lesne, S. E. , Sherman, M. A. , Grant, M. , Kuskowski, M. , Schneider, J. A. , Bennett, D. A. , & Ashe, K. H. (2013). Brain amyloid‐beta oligomers in ageing and Alzheimer's disease. Brain, 136, 1383–1398. 10.1093/brain/awt062 23576130 PMC3634198

[acel12978-bib-0024] Li, W. , Wu, Y. , Min, F. , Li, Z. , Huang, J. , & Huang, R. (2010). A nonhuman primate model of Alzheimer's disease generated by intracranial injection of amyloid-beta42 and thiorphan. METAB BRAIN DIS. 25, 277–284. 10.1007/s11011-010-9207-9. 20838863

[acel12978-bib-0025] Maezawa, I. , Zimin, P. I. , Wulff, H. , & Jin, L. W. (2011). Amyloid‐beta protein oligomer at low nanomolar concentrations activates microglia and induces microglial neurotoxicity. Journal of Biological Chemistry, 286, 3693–3706. 10.1074/jbc.M110.135244 20971854 PMC3030372

[acel12978-bib-0026] Oikawa, N. , Kimura, N. , & Yanagisawa, K. (2010). Alzheimer‐type tau pathology in advanced aged nonhuman primate brains harboring substantial amyloid deposition. Brain Research, 1315, 137–149. 10.1016/j.brainres.2009.12.005 20004650

[acel12978-bib-0027] Querfurth, H. W. , & LaFerla, F. M. (2010). Alzheimer's disease. New England Journal of Medicine, 362, 329–344. 10.1056/NEJMra0909142 20107219

[acel12978-bib-0028] Rodrigue, K. M. , Kennedy, K. M. , & Park, D. C. (2009). Beta‐amyloid deposition and the aging brain. Neuropsychology Review, 19, 436–450. 10.1007/s11065-009-9118-x 19908146 PMC2844114

[acel12978-bib-0029] Sanchez‐Mejias, E. , Navarro, V. , Jimenez, S. , Sanchez‐Mico, M. , Sanchez‐Varo, R. , Nuñez‐Diaz, C. , … Vitorica, J. (2016). Soluble phospho‐tau from Alzheimer's disease hippocampus drives microglial degeneration. Acta Neuropathologica, 132, 897–916. 10.1007/s00401-016-1630-5 27743026 PMC5106501

[acel12978-bib-0030] Savage, M. J. , Kalinina, J. , Wolfe, A. , Tugusheva, K. , Korn, R. , Cash‐Mason, T. , … McCampbell, A. (2014). A sensitive Aβ oligomer assay discriminates Alzheimer's and aged control cerebrospinal fluid. Journal of Neuroscience, 34, 2884–2897. 10.1523/JNEUROSCI.1675-13.2014 24553930 PMC6608513

[acel12978-bib-0031] Selkoe, D. J. , & Hardy, J. (2016). The amyloid hypothesis of Alzheimer's disease at 25 years. EMBO Molecular Medicine, 8, 595–608. 10.15252/emmm.201606210. 27025652 PMC4888851

[acel12978-bib-0032] Shankar, G. M. , Li, S. , Mehta, T. H. , Garcia‐Munoz, A. , Shepardson, N. E. , Smith, I. , … Selkoe, D. J. (2008). Amyloid‐beta protein dimers isolated directly from Alzheimer's brains impair synaptic plasticity and memory. Nature Medicine, 14, 837–842. 10.1038/nm1782 PMC2772133 18568035

[acel12978-bib-0033] Spittau, B. (2017). Aging microglia‐phenotypes, functions and implications for age‐related neurodegenerative diseases. Frontiers in Aging Neuroscience, 9, 194 10.3389/fnagi.2017.00194 28659790 PMC5469878

[acel12978-bib-0034] Steinerman, J. R. , Irizarry, M. , Scarmeas, N. , Raju, S. , Brandt, J. , Albert, M. , … Stern, Y. (2008). Distinct pools of beta‐amyloid in Alzheimer disease‐affected brain: A clinicopathologic study. Archives of Neurology, 65, 906–912. 10.1001/archneur.65.7.906 18625856 PMC2586606

[acel12978-bib-0035] Wang, J. , Dickson, D. W. , Trojanowski, J. Q. , & Lee, V. M. (1999). The levels of soluble versus insoluble brain Aβ distinguish Alzheimer's disease from normal and pathologic aging. Experimental Neurology, 158, 328–337. 10.1006/exnr.1999.7085 10415140

[acel12978-bib-0036] Xia, X. , Jiang, Q. , McDermott, J. , & Han, J. J. (2018). Aging and Alzheimer's disease: Comparison and associations from molecular to system level. Aging Cell, 17, e12802 10.1111/acel.12802 29963744 PMC6156542

[acel12978-bib-0037] Zhao, Q. , Lu, J. , Yao, Z. , Wang, S. , Zhu, L. , Wang, J. , & Chen, B. (2017). Upregulation of Aβ42 in the brain and bodily fluids of rhesus monkeys with aging. Journal of Molecular Neuroscience, 61, 79–87. 10.1007/s12031-016-0840-6 27647310

[acel12978-bib-0038] Zolochevska, O. , Bjorklund, N. , Woltjer, R. , Wiktorowicz, J. E. , & Taglialatela, G. (2018). Postsynaptic proteome of non‐demented individuals with Alzheimer's disease neuropathology. Journal of Alzheimer's Disease, 65, 659–682. 10.3233/JAD-180179 PMC6130411 30103319

[acel12978-bib-0039] Zolochevska, O. , & Taglialatela, G. (2016). Non‐Demented Individuals with Alzheimer's disease neuropathology: Resistance to cognitive decline may reveal new treatment strategies. Current Pharmaceutical Design, 22, 4063–4068. 10.2174/1381612822666160518142110 27189599 PMC6007878

