# Supplementary figures and images for: Brains of rhesus monkeys display Aβ deposits and glial pathology while lacking Aβ dimers and other Alzheimer's pathologies
Source: Aging Cell. 2019 Jun 4;18(4):e12978. doi: 10.1111/acel.12978 (PMC6612634; doi:10.1111/acel.12978)

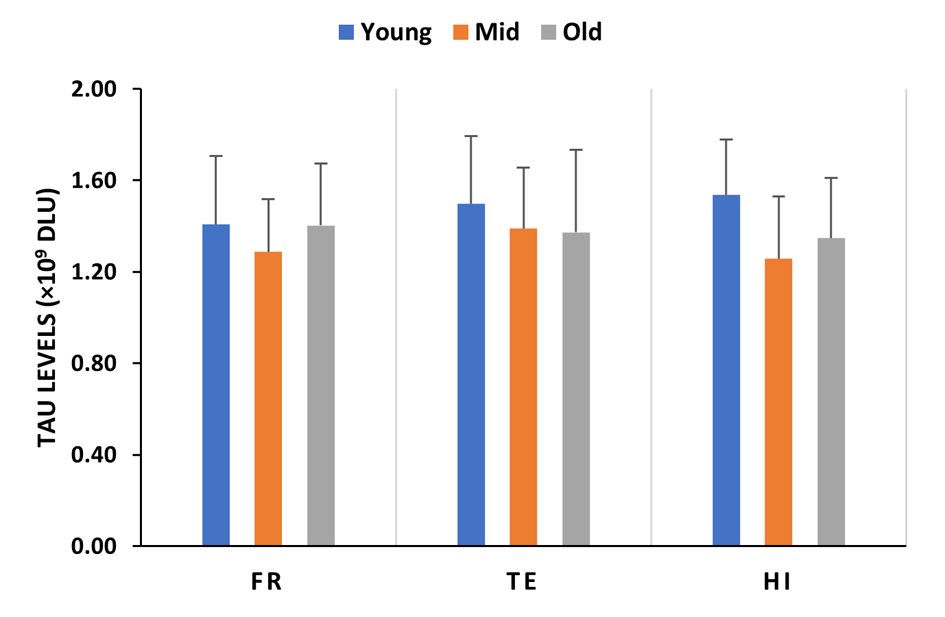

Supplement: Supplementary file 1 [file ACEL-18-e12978-s001.tif]

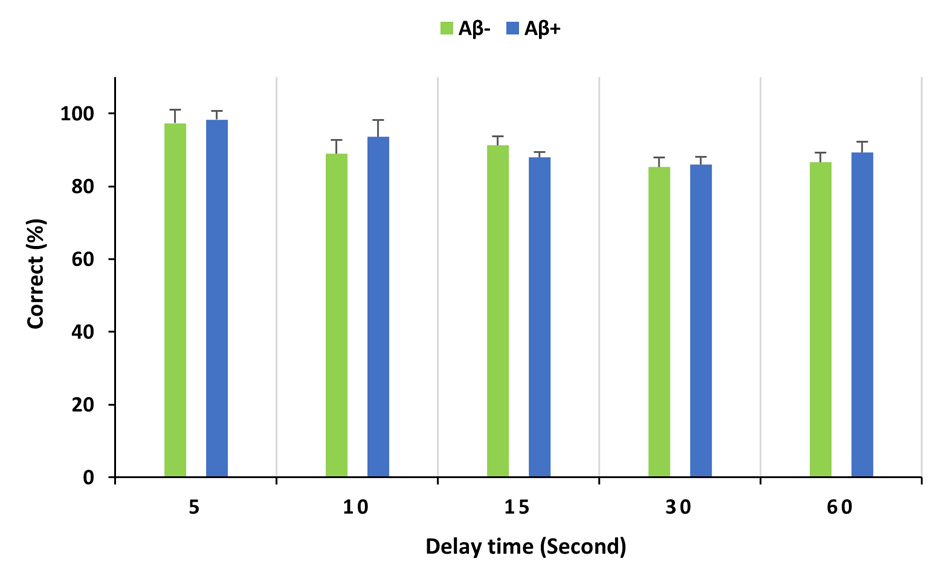

Supplement: Supplementary file 2 [file ACEL-18-e12978-s002.tif]
